# New-Onset Movement Disorders Associated with COVID-19

**DOI:** 10.5334/tohm.595

**Published:** 2021-07-08

**Authors:** Pedro Renato P. Brandão, Talyta C. Grippe, Danilo A. Pereira, Renato P. Munhoz, Francisco Cardoso

**Affiliations:** 1 Neuroscience and Behavior Lab, University of Brasília and Neurology Unit, Hospital Sírio-Libanês (Brasília), Brasília, DF, Brazil; 2 School of Medicine, Centro Universitário de Brasília (UNiCEUB), Brasília, DF, Brazil; 3 Neurology Unit, Instituto Hospital de Base do Distrito Federal (IHBDF), Brasília, DF, Brazil; 4 Brazilian Institute of Neuropsychology and Cognitive Sciences (IBNeuro), Brasília, DF, Brazil; 5 Toronto Western Hospital, Movement Disorders Centre, Toronto Western Hospital – UHN, Division of Neurology, University of Toronto, Toronto, Canada; 6 Movement Disorders Unit, Internal Medicine Department, Universidade Federal de Minas Gerais, Belo Horizonte, MG, Brazil

**Keywords:** movement disorders, parkinsonism, myoclonus, ataxia, opsoclonus, chorea, COVID-19, SARS-CoV-2

## Abstract

**Introduction:**

Movement disorders are increasingly described in hospitalized and milder cases of SARS-CoV-2 infection, despite a very low prevalence compared to the total patients.

**Methods:**

We reviewed the scientific literature published in English, spanning from the initial descriptions of COVID-19 until January 25, 2021, in the PubMed/MEDLINE database.

**Results:**

We identified 93 new-onset movement disorders cases (44 articles) from 200 papers screened in the database or reference lists. Myoclonus was present in 63.4% (n = 59), ataxia in 38.7% (n = 36), action/postural tremor in 10.8% (n = 10), rigid-akinetic syndrome in 5.38% (n = 5), oculomotor abnormalities in 20.4% (n = 19), catatonia in 2.1% (n = 2), dystonia in 1.1% (n = 1), chorea in 1.1% (n = 1), functional (psychogenic) movement disorders in 3.2% (n = 3) of the reported COVID-19 cases with any movement disorder. Encephalopathy was a common association (n = 37, 39.78%).

**Discussion:**

Comprehensive neurophysiological, clinical, and neuroimaging descriptions of movement disorders in the setting of SARS-CoV-2 infection are still lacking, and their pathophysiology may be related to inflammatory, postinfectious, or even indirect mechanisms not specific to SARS-CoV-2, such as ischemic-hypoxic brain insults, drug effects, sepsis, kidney failure. Cortical/subcortical myoclonus, which the cited secondary mechanisms can largely cause, seems to be the most common hyperkinetic abnormal movement, and it might occur in association with encephalopathy and ataxia.

**Conclusion:**

This brief review contributes to the clinical description of SARS-CoV-2 potential neurological manifestations, assisting clinical neurologists in identifying features of these uncommon syndromes as a part of COVID-19 symptomatology.

**Highlights:**

– Movement disorders are probably uncommon neurological manifestations in SARS-CoV-2 infection;
– Myoclonus is the most reported movement disorder associated with COVID-19, its clinical complications or pharmacological management;
– The pathophysiology is yet not well-understood but can include systemic inflammation, autoimmune mechanisms, or hypoxia.

## 1. Background

Coronavirus disease 2019 (COVID-19) has been associated with various neurological symptoms [1,2]. Numerous putative dissemination mechanisms of the central nervous system (CNS) were hypothesized, including direct retrograde spread through the olfactory nerve via the transcribiform pathway or hematogenous spread via the blood-brain barrier. The neuronal damage could happen through different but not mutually exclusive mechanisms, including direct viral insult, cytokine release syndrome, hypoxia, immune-mediated neuroinflammation (post or para-infectious), coagulopathy, and endothelial dysfunction syndrome [1]. Headache, altered mental status (encephalopathy), chemosensory dysfunction (anosmia, ageusia), cerebrovascular events, and myalgia seem to be the most typical neurological manifestations [2]. However, the description of the isolated virus in the cerebrospinal fluid (CSF) is seemingly rare, suggesting that direct viral meningoencephalitis is not usual.

Movement disorders are increasingly being described not only among hospitalized patients but also in milder cases of SARS-CoV-2 infection, despite a possible very low prevalence in comparison to the total cases [3,4]. This study aims to summarize and describe, through a systematic procedure, relevant clinical and ancillary exam findings in patients with new-onset movement disorders associated with COVID-19.

## 2. Methods

### 2.1 Literature Search and Review

**Figure 1:**
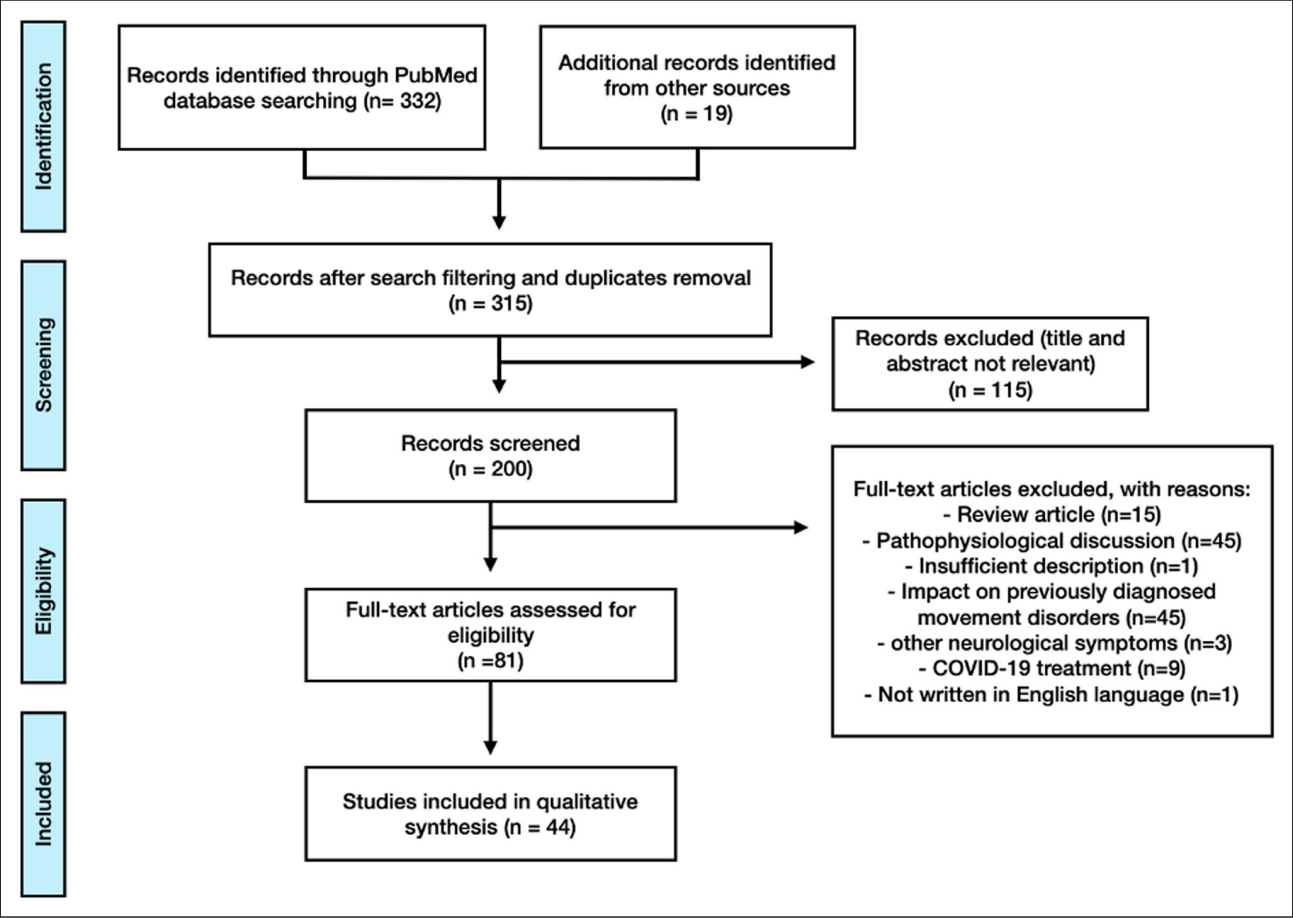
Prisma flowchart for a systematic review of articles on new-onset movement disorders and COVID-19 from PubMed database on January 25, 2021. Search criteria used: “COVID-19” OR “SARS-CoV-2” OR “Coronavirus Disease 2019” OR “2019 n-CoV” OR “2019 Novel Coronavirus” AND (“movement disorders” OR “myoclonus” OR “ataxia” OR “parkinsonism” OR “Parkinson’s disease” OR “chorea” OR “dystonia” OR “myoclonus” OR “catatonia” OR “tremor”).

We first searched the PubMed/MEDLINE database for relevant literature published in the English language, between the initial descriptions of COVID-19 (December 31, 2019) and January 25, 2021, using the search terms “COVID-19” OR “SARS-CoV-2” OR “Coronavirus Disease 2019” OR “2019 n-CoV” OR “2019 Novel Coronavirus” AND (“movement disorders” OR “myoclonus” OR “ataxia” OR “parkinsonism” OR “Parkinson’s disease” OR “chorea” OR “dystonia” OR “myoclonus” OR “catatonia” OR “tremor”)” as search terms (n = 332). We added filters for Case Reports, Classical Articles, Clinical Studies, Clinical Trial, Comparative Studies, Original Journal Articles, Letters, Observational Studies, Human Species and identified 296 records for screening (titles/abstracts). We conducted a prospection in the reference list of selected papers and other sources, identifying additional 19 items. After excluding 115 records with abstracts and titles irrelevant to this study’s scope, we screened 200 records for eligibility. We evaluated 81 full-text papers for eligibility after withdrawing 119 articles for the following reasons: they were not written in English or were focused on social or healthcare impact of COVID-19 in patients already diagnosed with a movement disorder, dealt with aggravation of pre-existing movement disorders, telemedicine management strategies, or were listed as review/hypothesis/opinion articles (***Figure 1***). A total of 44 studies were included for qualitative and quantitative synthesis as representative of neurological descriptions of new-onset movement disorders in the context of SARS-CoV-2 infection.

### 2.2 Data extraction, synthesis, and analysis

Two authors (PB and TG) collected data independently, using a predefined protocol. One of the five authors (PB) checked the extracted data, and discrepancies were resolved by consensus between the two data collectors. Variables of interest included counts for single or combined movement disorders (myoclonus, dystonia, ataxia, parkinsonism, chorea, catatonia, action/postural tremors, oculomotor disturbances), associated clinical features (encephalopathy, stroke), summaries of ancillary exam results (FDG-PET, MRI/CT, ENMG, CSF, and EEG). The attempted treatment options (symptomatic treatment or immunotherapy, such as intravenous immunoglobulin – IVIg, steroids) were also counted. Clinical characteristics were quantified as case counts and ratios of case counts to total cases. Symptoms not initially reported in individual studies were considered absent (zero) rather than missing (NA).

Descriptive statistical analysis was performed using Wizard v2.0.4 (Evan Miller, 2020, *https://www.wizardmac.com*) and R v4.0.4 (*https://www.R-project.org/*). A world map including the reported cases was generated using Datawrapper (*https://app.datawrapper.de*). The “brms” R package was used to obtain the parameter values’ Bayesian posterior distribution [5]. “Brms” uses the logit-link to convert the linear predictor zi (zero-inflated) to a probability. The logit-link accepts values between 0 and 1 and returns values on the real line. As a result, it enables the conversion of probabilities to linear predictors. Samples were drawn using No-U-Turn-Sampler (NUTS) sampling, the default algorithm used by the Stan program [6]. Rhat was used as a potential scale reduction factor on split chains (at convergence, Rhat = 1). We fit the model using four Hamiltonian Monte-Carlo (HMC) chains. We used Jeffrey’s beta (0.5, 0.5) as the prior argument for zi rather than the default “flat” beta (1, 1) used by “brms”. The intercept parameter prior was normal (0, 10). The Bayesian plot was created using the “bayesplot” R package (*https://mc-stan.org/bayesplot/*) [7].

Using counts as the measure of movement disorders frequency (main variables extracted from the articles), we performed an unsupervised grouping procedure (clustering) to separate and observe co-occurrence between the symptoms in the reported articles. The “NbClust” R package was used to determine the optimal number of clusters [8]. It provides 30 indices for that task and proposes the best clustering scheme by varying all combinations of several clusters, distance measures, and clustering methods (Supplementary Figure 1). We then employed a random forest (RF) clustering strategy to avoid variable transformation (e.g., categorical features). By forming two unique groups (as determined by the NbClust’s method), RF clustering separated the original data from the virtual clusters. The similarity between two data instances was assessed using the proportion of trees that shared a single leaf node. The PAM (partition around medoids) technique [9] was used to assign each object to the closest medoid (object with the smallest dissimilarity) as a step to create the clusters.

## 3. Results

### 3.1 Studies characteristics

Forty-four initial papers were ultimately chosen from a total of 81 full-text articles that were evaluated for eligibility, including a total of 93 specific cases of movement disorders caused by or acutely linked to SARS-CoV-2 infection or its pharmacological treatment attempts. ***Table 1*** summarizes these cases and publications.

**Table 1:** Comprehensive description of COVID-19 cases presenting with movement disorders phenomenology.


AUTHORS, YEAR, COUNTRY, STUDY DESIGN	CLINICAL SCENARIO, CASE COUNT, METHOD, SEVERITY	MOV. DISORDERS PRESENTATION	NEUROIMAGING	CSF OR RELEVANT LAB STUDIES	NEUROPHYSIOLOGICAL STUDIES	TREATMENT

Romero-Sánchez et al., [3], Spain, Retr., Obs.,	Hospital setting, 6 cases, RT-qPCR or serology	6 cases: hyperkinetic mov.. 3 patients w/ mostly myoc. tremor and 3 w/ tardive synd. w/ oromandibular dyskinesia and tremor (related to neuroleptics)	N/A	N/A	N/A	N/A

Studart-Neto et al., [4], Brazil, Retr., Obs.	Hospital setting, 6 cases, RT-qPCR, 2 inpatient, 4 ICU – mechanical ventilation	6 cases: hyperkinetic mov. (myoc.); 4 of those w/ encephalopathy and one w/ a cerebrovascular disorder	N/A	N/A	N/A	N/A

Faber et al., [10], Brazil, Case report	Hospital setting, 1 case, RT-qPCR, Inpatient	Anosmia and acute levodopa-responsive parkinsonism	**FDG-PET:** Normal glucosemetab. in **MRI (3T)**, normal neuromelanin and nigrosome1 imaging; **TRODAT-1 SPECT:** Nigrostriatal denervation	Unremarkable	N/A	levodopa-responsive

Méndez-Guerrero et al., [11], Spain, Case report	Hospital setting, 1 case, RT-qPCR, ICU w/ mechanical ventilation	Hyposmia, generalized rest and postural myoc., fluctuating consciousness, opsoclonus, and an asymmetric parkinsonian synd.	**DaT-SPECT:** Bilat. decrease in presynaptic dopamine uptake asymmetrically involving both putamina; **CT:** unremarkable **MRI:** unremarkable	Unremarkable	**EEG:** diffuse mild and reactive slowing	Levetiracetam, not-responsive to apomorphine

Piscitelli et al. [12] Italy, Case report	Hospital setting, 1 case, RT-qPCR, Inpatient	Functional tremor w/ entrainment phenomenon and distractibility	Normal brain and spine **MRI**	N/A	**SSEP:** normal in legs	Supportive

Mas Serrano et al., [13], Spain, Case report	Hospital setting, 2 cases, RT-qPCR, Inpatient	Serotonergic synd. (w/ myoc.) due to association of lopinavir/ritonavir and psychotropic drugs (duloxetine, lithium, risperidone, haloperidol, morphine)	**Case 1:** normal MRI **Case 2:** normal CT	High levels of CPK.	**EEG** w/ encephalopathic findings (both cases)	**Case 1:** cyproheptadine **Case 2:** clonazepam

Rábano-Suárez et al., [14], Spain, Case series	Hospital setting, 3 cases (2 w/ a typical clinical scenario and lung CT scan, 1 RT-qPCR), 1 ICU and mechanical ventilation, 2 inpatient	Generalized myoc. and hypersomnia	**Case 1, 3:** normal MRI, **Case 2:** normal head CT;	**Case 1:** Unremarkable **Case 2, 3:** not reported	**EEG:** mild background slowing (3)	3 cases w/ CS (MP), 2 cases w/ clonazepam and levetiracetam, 1 case w/ valproic acid

Cuhna et al. [15], France, Case series	Hospital, 5 cases, RT-qPCR, ICU w/ mechanical ventilation	3 subjects w/ act. and postural tremor in UL, one patient w/ hemicorporal act. tremor, one w/ jerky tremor; All w/ mild motor deficit	**MRI:** microbleeds (4), unilateral nigrossomal abnormality (1) **SPECT:** frontotemporal hypoperfusion (1) **DAT-Scans:** normal (4).	N/A	**EEG/ENMG:** cortical/subcortical myoc. (2), short myoclonic bursts.	N/A

Paterson et al., [16], United Kingdom, Retr., Obs.	Hospital, 2 cases RT-qPCR, Inpatient	**Case 1:** encephalopathy case w/ act. tremor and LL ataxia; **Case 2:** opsoclonus, stimulus sensitive myoc., hyperekplexia, and convergence spasm	**Case 1:** neuroimaging within normal limits; **Case 2:** normal Brain MRI;	**Case 2:** unremarkable	**Case 2: EEG:** unremarkable	**Case 1:** supportive; **Case 2:** steroids, levetiracetam, clonazepam

Chaumont et al. [17], France, Case series	Hospital 4 cases, RT-qPCR, ICU w/ mechanical ventilation	Mixed central and peripheral features: encephalopathy (4), ataxia (4), postural and act. myoc. (4), and polyneuropathy (4)	**MRI:** 3 cases w/ normal brain imaging, one w/ recent stroke in MCA territory;	Mildly elevated protein levels (2)	**EEG:** background slowing (3); normal (1). **ENMG:** motor demyelinating polyradiculoneuropathy (3) or diffuse lower motor neuron involvement (1)	IVIg 0.4 g/kg (4), steroids (3)

Dijkstra et al. [18], Belgium, Case report	Hospital, 1 case, RT-qPCR, Inpatient	Action-induced myoclonic jerks, stuttering speech hyposmia, transient ocular flutter, gait ataxia, attention and memory deficits, hypervigilance, and insomnia.	Normal brain and spinal **MRI** **PET-CT:** neg. screening for neoplasia.	Unremarkable, neg. autoimmune and paraneoplastic antineuronal Abs	N/A	CS and IVIg

Gutiérrez-Ortiz et al. [19], Spain, Case report	Hospital, 2 cases, RT-qPCR, Inpatient	Anosmia, ageusia, right internuclear ophthalmoparesis, right fascicular oculomotor palsy, ataxia, areflexia, (Miller-Fisher synd.)	**Head CT:** normal (2)	Pos. GD1b-IgG (1), albuminocytologic dissociation (2), mildly elevated CSF protein levels.	N/A	**Case 1:** IVIg **Case 2:** supportive, spontaneous recovery

Lantos et al., [20], USA, Case report	Hospital, 1 case, RT-qPCR, Inpatient	Progressive ophthalmoparesis (left CN III and Bilat. CN VI palsies), ataxia, and hyporeflexia (Miller-Fisher synd.)	**MRI:** prominent enhancement w/ gadolinium, T2 hyperintense left oculomotor nerve (CN III)	Neg. GD1b-IgG,	N/A	IVIg

Balestrino et al., [21], Italy, Case report	Hospital, 1 case, RT-qPCR, Inpatient	Asthenia, gait ataxia, balance impairment, confusion, and drowsiness	**Head CT:** Unremarkable	N/A	**EEG:** focal delta slowing, sporadic spikes	Lopinavir/ritonavir, chloroquine, CS and levofloxacin

Mao et al. [22], China, Retr., Obs.	Hospital, 1 case, RT-qPCR, ICU w/ mechanical ventilation	Ataxia and severe COVID-19 infection	N/A	N/A	N/A	N/A

Fadakar et al., [23], Iran, Case report	Hospital, 1 case, RT-qPCR, Inpatient	Myalgia, progressive vertigo, headache, dysarthria, and cerebellar ataxia.	**MRI:** edema of cerebellar hemispheres and vermis, leptomeningeal enhancement.	Mild lymphocytic pleocytosis, elevated protein, and lactate dehydrogenase and pos. for SARS-CoV-2, Neg. antineuronal Abs panel	N/A	Lopinavir/ritonavir

Xiong et al. [24], China, Retr., Obs.	Hospital, 2 cases, RT-qPCR Inpatient	Tics/tremor (functional?). Case 1: 8 days after initial symptoms Case 2: 41 days after initial symptoms	N/A	N/A	N/A	N/A

Klein et al. [25], USA, Case report	Hospital, 1 case, diagnostic method not available, Inpatient	Bilat. intention tremor and wide-based gait (ataxia)	**Head CT** and **angioCT:** no abnormalities; Brain **MRI:** no acute findings	N/A	N/A	Propranolol

Cohen et al., [26], Israel, Case report	Hospital, 1 case, RT-qPCR, Inpatient	Hypomimia and hypophonia. Cogwheel rigidity in neck, right arm, left arm, Asymmetric bradykinesia, no tremor. Slow gait, no right arm swing. No postural instability.	**MRI:** unremarkable **¹**⁸**F-DOPA PET:** asymmetrically decreased uptake in putamens, more apparent on the left side.	Unremarkable, Neg. for GABA type B receptors, NMDAR, CASPR2, AMPA receptor type 1, AMPA receptor type 2, and LGI1.	**EEG:** unremarkable	Low-dose pramipexol

Sanguinetti & Ramdhani., [27], USA, Case report	Hospital, 1 case, RT-qPCR, Inpatient	Appendicular and axial ataxia, act. myoc., act. tremor, Spontaneous horizontal and vertical eye oscillations (opsoclonus-myoclonus-ataxia synd.)	**Brain MRI-** normal	N/A	N/A	CS and IVIG (400mg/kg/d, 5d)

Ros-Castelló et al. [28], Spain, Case report	Hospital, 1 case, RT-qPCR ICU w/ mechanical ventilation	Myoc. in UL and neg. myoc. in LL, leading to falls, delayed onset	**Brain MRI:** cortical and brainstem ischemic lesions (hyperintensities in DWI and FLAIR)	N/A	N/A	Low-dose clonazepam

Wright et al., [29], New Zealand, Case report	Hospital, 1 case, RT-qPCR, Inpatient	Saccadic oscillations (ocular flutter and opsoclonus) and gait ataxia, w/ no myoc., encephalopathy	**Brain MRI:** chronic white matter changes	N/A	N/A	Supportive, deceased

Muccioli et al. [30], Italy, Case report	Hospital, 1 case, RT-qPCR, ICU w/ mechanical ventilation	Multifocal myoc. elicited by act. and tactile stim., predominant in the right proximal LL	**Brain MRI:** chronic white matter changes	Mild pleocytosis (5 cells/μL), elevated protein (75mg/dL), neg. SARS-CoV2 PCR, elevated IL-8. Neg. antineuronal Abs.	**EEG:** normal; **PolyEMG:** multifocal positive myoc. w/ a burst of 140 -220 milliseconds **EEG-EMG back-averaging:** no jerk-locked discharges.	Levetiracetam and clonazepam

Borroni et al., [31], Italy, Case series	Hospital, 2 cases, RT-qPCR, Inpatient	Diaphragmatic myoc. (jerky contractions of abdominal muscles, diaphragm);	**Case 1:** **Brain MRI:** normal **Case 2:** **Head CT:** normal;	**Case 1:** mild pleocytosis (8 cells/mm3). **Case 2:** lymphocytosis (24 cell/mm3), increased protein (46 mg/dL).	**Case 1: EMG:** 3 Hz synchronous discharges **EEG:** normal; **SSEP:** normal **Case 2: EEG:** synchronous and asynchronous LPDs (w/ myoc.);	**Case 1:** low dose clonazepam **Case 2:** levetiracetam

Schellekens et al., [32], Netherlands, Case report	Outpatient, 1 case, RT-qPCR, Inpatient	Generalized myoc. jerks of trunk, face, and limbs, particularly UL, at rest, worsened w/ posture and action. Cerebellar ataxia.	**Brain MRI:** unremarkable.	Normal routine profile; neg. for Paraneoplastic antineuronal Abs.	N/A	Levetiracetam

Anand et al., [33], USA, Case series	Hospital, 8 cases, RT-qPCR, 7 ICU w/ mechanical ventilation, 1 inpatient	Stimulus- or action-induced myoc. (7) and spontaneous myoc. (1). Generalized (5) and UL myoc. (3).	**Head CT**s: unremarkable (5) **MRI:** unspecific temporal T2 hyperintensity (1) **MRI:** Diffuse pachymeningeal enhancement (1)	Normal protein (2), High protein levels (83 mg/dL) (1), normal cell count (3)	**EEG:** Bifrontal sharp waves (1), background slowing (2)	Levetiracetam (3), Ketamine (1), dexmedetomidine (5), midazolam (1), lorazepam (3), primidone (1), clonazepam (1), valproic acid (3)

Grimaldi et al., [34], France, Case report	Hospital, 1 case, RT-qPCR, Inpatient	Act. tremor, cerebellar ataxia, stimulus-sensitive and spontaneous diffuse myoc.	**Brain MRI:** Unremarkable **¹**⁸**F-FDG PET:** Putaminal and cerebellum hypermetab., diffuse cortical hypometab.	Normal cell count, mildly elevated protein level (49 mg/dL), neg. RT-qPCR, and neg. OCBs. Immunostaining: Abs against nuclei of Purkinje, striatal and hippocampal neurons.	**EEG:** background slowing, reactive to stimulation (1)	IVIg, IV CS, Low-dose clonazepam

Byrnes et al., [35], USA, Case report	Hospital, 1 case, RT-qPCR, Inpatient	Homeless and drug-addicted. Encephalopathy and choreiform mov.	**Brain MRI:** Multiple focal enhancing lesions: Bilat. putamen and cerebellum. Several cortical and subcortical lesions, hippocampus, right basal ganglia.	Mildly lymphocytic pleocytosis and increased myelin basic protein.	N/A	IV CS, IVIg, oral CS Chorea improved on day 15, w/ an immediate resolution on day 22.

Kopscik et al. [36], USA, Case report	Hospital, 1 case, RT-qPCR and serology, Inpatient	Multiple cranial nerve abnormalities, dysmetria, sensory ataxia, and absent LL reflexes	**Brain MRI:** unremarkable	Anti-GQ1b IgG Abs (1:100), w/ lymphocytic predominance, normal protein	N/A	Convalescent plasma, tocilizumab, and intravenous immunoglobulin.

Fernández-Domínguez et al., [37], Spain, Case report	Hospital, 1 case, RT-qPCR and serology, Inpatient	LL areflexia, sensory gait ataxia.	**Brain MRI:** unremarkable	Increased protein level (110 mg/dl) Normal cell count. Neg. antiganglioside Abs. SARS-CoV-2 neg..	**EMG:** slight F-wave delay in ULs. **Visual evoked potential:** unremarkable	IVIg, w/ improv..

Dinkin et al., [38], USA, Case report	Hospital, 1 case, RT-qPCR, Inpatient	Partial unilat. oculomotor palsy, Bilat. abducens palsies. LL hyporeflexia and hypesthesia, and gait ataxia.	**Brain MRI:** enhancement, T2-hyperintensity, and enlargement of oculomotor nerve	Ganglioside Abs: neg.	**N/A**	IVIg, w/ improv..

Perrin et al., [39], France, Retr., Obs.	Hospital, 5 cases, RT-qPCR, ICU, 2 w/ mechanical ventilation	Confusion (n = 5), tremor (n = 5), cerebellar ataxia (n = 4), behavioral alterations (n = 5), aphasia (n = 4), pyramidal synd. (n = 4), coma (n = 2), cranial nerve palsy (n = 1),	**Brain MRI:** acute leukoencephalitis (cases 1, 2, and 4), microbleeds in the corpus callosum (1); cytotoxic edema mimicking stroke (case 2), and normal results (cases 3 and 5);	SARS-CoV-2 PCR: neg.. CSF/serum albumin index increased (3), Normal cell count (5), Absent IgG intrathecal synthesis (5), OCBs (3), Antineuronal Abs absent (5).	**Case 1:** EEG: asymmetric slow-wave spikes and occipital focus **Case 2:** EEG: slow Bilat. delta bursts or predominant opposite bifrontal diversions w/ Bilat. 5–6 Hz theta **Case 3:** normal	Case 1: CS (DM) Case 2: CS (DM) + IVIg w/ improv. Case 3, 4: spontaneous recovery Case 3: CS (DM + MP) w/ improv.

Hayashi et al., [40], Japan, Case report	Hospital, 1 case, RT-qPCR, ICU	Marked dysmetria and mild ataxic gait	**Brain MRI:** hyperintensity in splenium of corpus callosum on DWI [mild encephalitis/encephalopathy w/ a reversible splenial lesion (MERS)]	N/A	N/A	Spontaneous recovery of neurologic symptoms

Khoo et al., [41], England, Case report	Hospital, 1 case, RT-qPCR, Inpatient	Myoc., ocular flutter, convergence spasm, hyperekplexia, confusion	**Brain MRI:** normal	Unremarkable. Neg. viral PCR panel. Neg. SARS-CoV-2 PCR. Neg. antineuronal Abs.	**EEG:** normal	Levetiracetam, clonazepam w/ partial improv. in myoc. and hyperekplexia. CS (MP and prednisone); all neurological symptoms improved

Pilotto et al., [42], Italy, Case report	Hospital, 1 case, RT-qPCR, Inpatient	Irritability, confusion, and asthenia. Severe akinetic synd. and mutism; Frontal release signs, nuchal rigidity.	**Head CT:** unremarkable **Brain MRI:** unremarkable	Lymphocytic pleocytosis (18/uL), increased protein (69.6 mg/dL). Neg. viral panel. Neg. SARS-CoV-2 PCR. Neg. OCBs and antineuronal Abs.	**EEG:** Generalized background slowing, decreased reactivity	CS (MP 1 g/day (five days), prednisone, w/ improv.

Delorme et al., [43], France, Case series	Hospital, 2 cases, RT-qPCR, Inpatient	**Case 1:** psychomotor agitation, cognitive/behavioral frontal synd., UL myoc., cerebellar ataxia. **Case 2:** psychomotor agitation, anxiety, depressed mood, dysexecutive and cerebellar synd. (hypotonia, gait ataxia, dysmetria, dysarthria, nystagmus)	**Brain FDG-PET:** Bilat. prefrontal and left parieto-temporal hypometab., hypermetab. in vermis **Brain FDG-PET:** bilat. Orbitofrontal hypometab., Bilat. Hypermetab. in striatum and vermis.	**CSF 1:** Mild pleocytosis (6 cells/mm3), normal protein. **CSF 2:** 0 cells/m3, normal protein.	**EEG:** normal in both cases	1: IVIg 2 g/kg. Gradual improv., up to 6 weeks long. 2: CS (2 mg/kg/day for 3 days). Improved cerebellar symptoms. Antidepressants (paroxetine and mirtazapine)

Caan et al., [44], USA, Case report	Hospital, 1 case, RT-qPCR, Inpatient	Hallucinations, delusions, apathy, muscle rigidity and diaphoresis, catatonia.	**Brain MRI:** unremarkable	Normal cell count, protein, and gluc. level.	N/A	Lorazepam with partial recovery

Pilotto et al., [45], Italy, Longitudinal, multicentric	Hospital, 25 encephalitis cases, 1 w/ a mov. disorder, RT-qPCR, Inpatient	Altered mental status w/ extrapyramidal synd. (parkinsonian synd.)	**Brain MRI:** frontal T2 hyperintensities	Cels.: 4/mm^3^; mildly elevated protein;	N/A	N/A

Povlow et al., [46], USA, Case report	Hospital, 1 case, RT-qPCR, Inpatient	Nausea, dysarthria, dysmetria, dysdiadochokinesia, mod. appendicular ataxia, unable to stand unassisted.	**Brain MRI:** unremarkable	Mild lymphocytic pleocytosis (7 cells/mm3), normal protein, neg. meningitis/encephalitis panel; neg. OCBs.	N/A	Spontaneous partial recovery

Franke et al., [47], Germany, Retrospective case series	Hospital, 8 cases w/ a mov. disorder, RT-qPCR, ICU – respiratory status not available	**Case 1:** nystagmus, orofacial myoc., delirium **Case 2:** nystagmus, generalized stimulus-sensitive myoc. **Case 3:** unilat. stimulus-sensitive myoc. **Case 4:** unilat. orofacial myoc. **Case 5:** Delirium, myoc., epileptic seizures **Case 6:** unilat. faciobrachial myoc., multifocal strokes **Case 7:** Oculomotor paresis, transient generalized myoc., prolonged awakening **Case 8:** Asym. Dystonia (UL), delirium	**Head CT:** Normal (4); **PET-FDG:** increased metab. in basal ganglia, limbic system, and cerebellum (1). **Brain MRI:** Marked fornix edema (1), right middle cerebral artery (MCA) ischemia (1)	N/A	N/A	N/A

Fernando et al., [48], Philippines, Case report	Hospital, 1 case, RT-qPCR, Inpatient	Bilat., asynchronous, irregular myoc. (UL, LL)	**Brain MRI:** unremarkable	N/A	N/A	Resolution 2 weeks after hydroxychloroquine withdraw

Deocleciano de Araujo et al., [49], Brazil, Case report	Hospital, 1 case, RT-qPCR, ICU	Disorganized behavior, social withdrawal, reduced motor output, body stiffness, negativism, refusal to feed, weight loss.	**Head CT:** normal	Protein 55 mg/dL	N/A	Lorazepam, sertraline, Electroconvulsive therapy, resolution after 50 days

Emamikhah et al., [50], Iran, Case series	Hospital, 7 cases, Rt-qPCR (5 cases), serology (1 case), clinical (1 case) Inpatient	Myoc. (7), opsoclonus (3), ataxia (7), voice tremor (6).	**Head CT:** normal **(2)**; **Brain MRI:** normal (4)	Normal cell count (3), protein (3), gluc. (3). Neg. OCBs (1).	**EEG:** normal (1).	Clonazepam (6), Levetiracetam (3), Valproate(5), IVIg (5), CS (1)

Urrea-Mendoza et al., [51], USA, Case report	Hospital, 1 case, RT-qPCR Inpatient	Opsoclonus, myoc., and ataxia	**Brain MRI:** normal	N/A	N/A	Clonazepam, valproate (divalproex), steroids


PET – positron emission tomography; SPECT – Single-photon emission computed tomography, CSF – cerebrospinal fluid, COVID-19 – Coronavirus Disease-2019, RT-qPCR – Quantitative reverse transcription PCR, UL – upper limb; MRI – magnetic resonance imaging, EEG – electroencephalogram; ENMG – electroneuromyography, DAT-Scan: dopamine transporter imaging; LL – lower limb; Abs – antibodies; MCA – middle cerebral artery; DWI – diffusion-weighted imaging; FLAIR – fluid-attenuated inversion recovery; SSEP – somatosensory evoked potential; LPDs – lateralized periodic discharges; DM – dexamethasone, MP – methylprednisolone, IVIg – Intravenous immunoglobulin, ICU – Intensive care unit; Gluc: glucose; Prot: protein; CS: corticosteroids; OCB: oligoclonal bands; Abs: antibodies; N/A: Not available. Retr. – retrospective, Obs. – observational, Myoc.- myoclonus, Bilat. – bilateral., Unilat. – unilateral, Pos. – positive, Neg. – negative.

**Figure 2:**
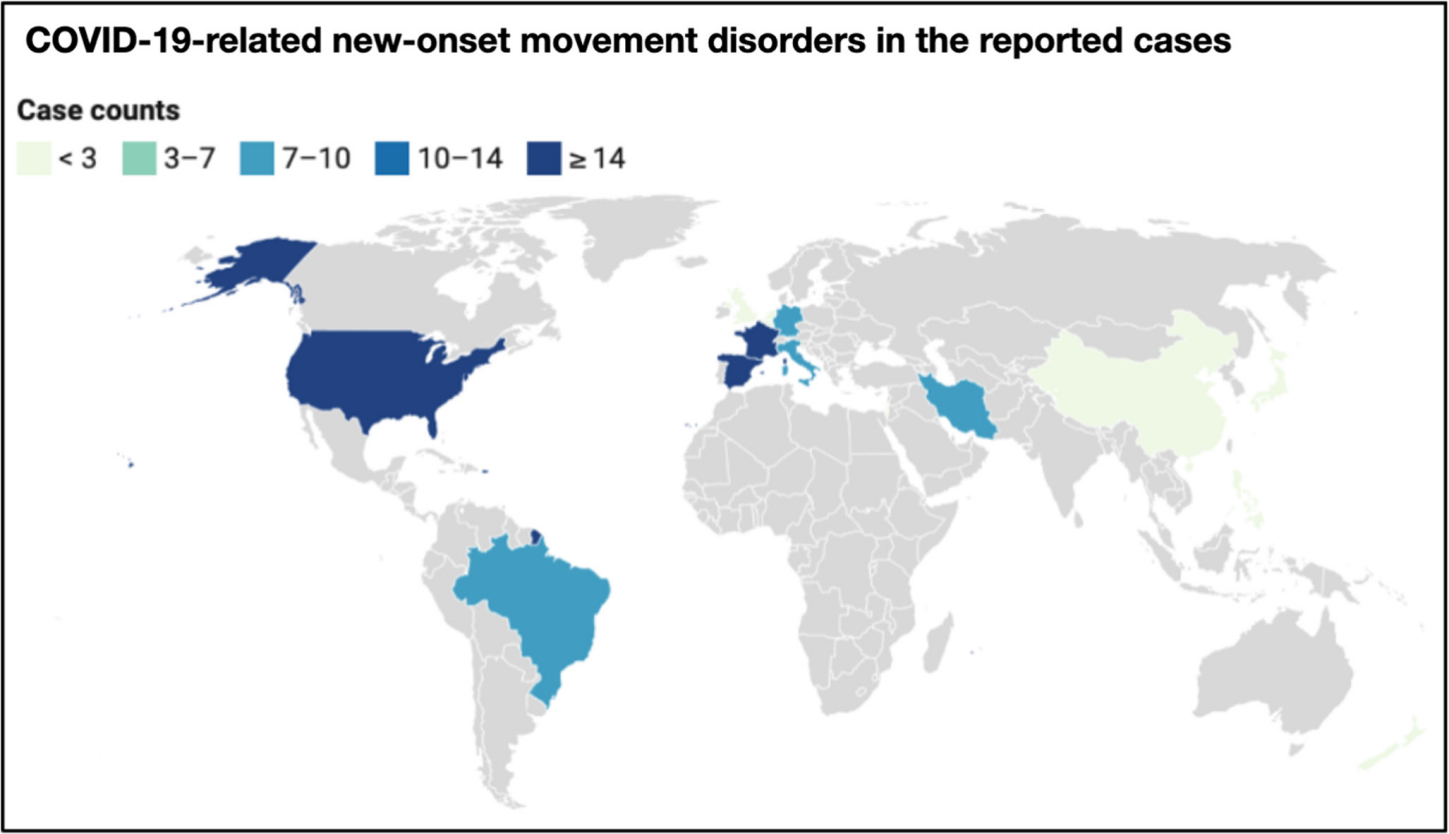
COVID-19-associated new-onset movement disorders: case counts in the reported original articles sample. A scale measuring total case counts ranges from 0 to 17 and is visually represented using a color scale ranging from light green to dark blue.

The cases came generally from regions where COVID-19 epidemics were on course. A proportion of 57.0% of the cases were reported in Europe (n = 53), 26.9% in the Americas (n = 25), 15% in Asia (n = 14), 1% in Oceania (n = 1). Most reported cases came from France (n = 17, 18.3%), United States (n = 17, 18.3%), Spain (n = 16, 17.2%), followed by Brazil (n = 8, 8.6%), Iran (n = 8, 8.6%) Germany (n = 8, 8.6%), Italy (n = 7, 7.5%), United Kingdom (n = 3, 3.2%) and China (n = 3, 3.2%). Japan, Netherlands, Israel, New Zealand, Philippines, and Belgium reported 1 case each (1.1% each), as represented in ***Figure 2***.

**Figure 3:**
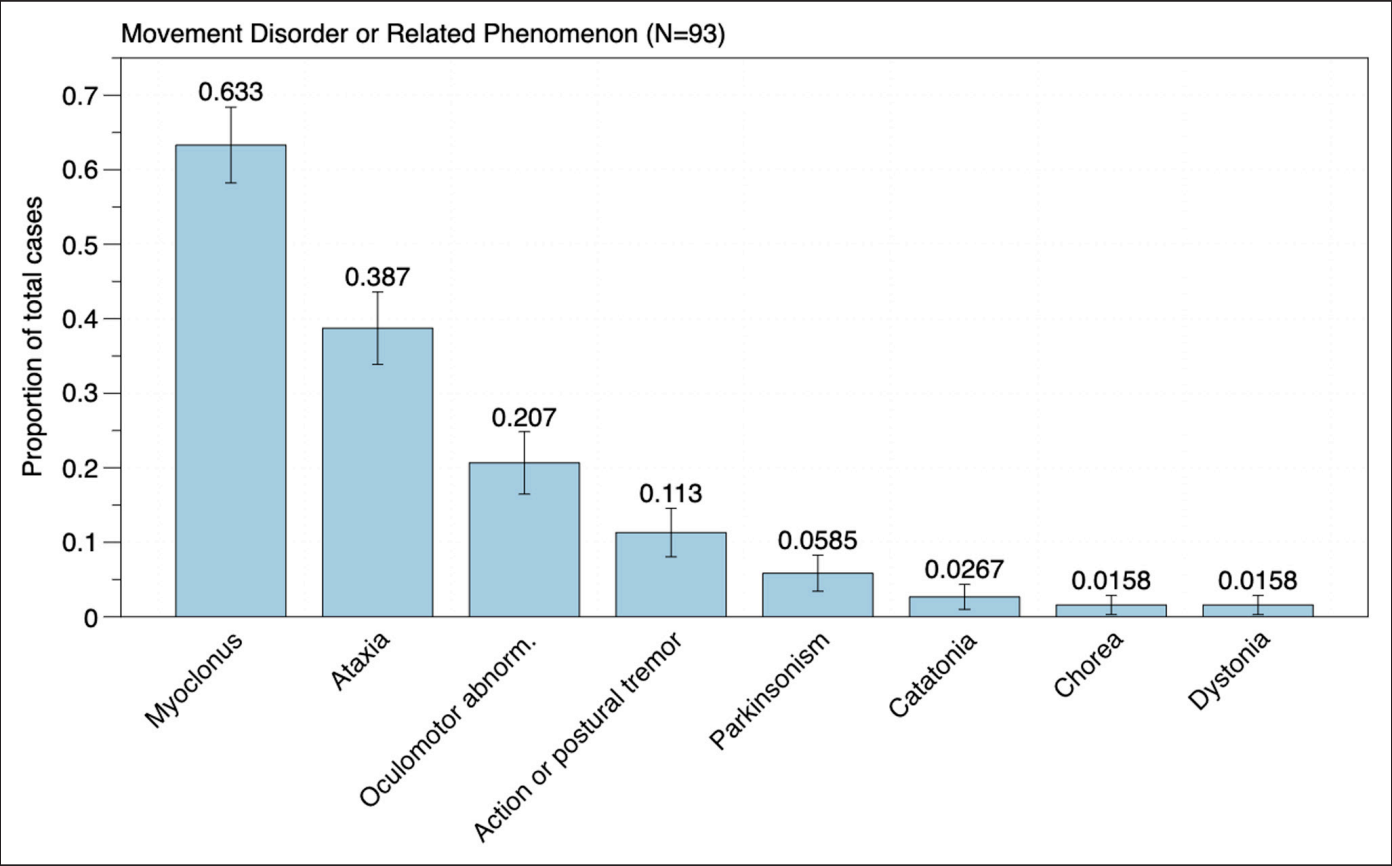
The bar plots’ numbers depict the Bayesian binomial posterior proportion of the described movement disorders or associated phenomenology to the total number of described cases (N = 93) in the reviewed articles. The error bar reflects the error in the posterior estimates. Data source: extracted from individual patients, original articles.

The major findings are depicted in ***Figure 3*** as Bayesian binomial posterior proportions. ***Table 1*** summarizes the clinical and ancillary exam data from each original study to serve as a simple reference guide for physicians and researchers.

### 3.2 Clinical characteristics

Of the 93 movement disorder cases identified in the literature search [3,4,10,11,12,13,14,15,16,17,18,19,20,21,22,23,24,25,26,27,28,29,30,31,32,33,34,35,36,37,38,39,40,41,42,43,44,45,46,47,48,49,50,51], myoclonus was present in 63.4% (n = 59), ataxia in 38.7% (n = 36), action/postural tremor in 10.8% (n = 10), rigid-akinetic syndrome (parkinsonism) in 5.38% (n = 5), oculomotor abnormalities (opsoclonus, ocular flutter) in 20.4% (n = 19), catatonia in 2.1% (n = 2), dystonia in 1.1% (n = 1), chorea in 1.1% (n = 1), functional (psychogenic) movement disorders in 3.2% (n = 3) of the reported COVID-19 cases with any movement disorder (***Figure 2***). Encephalopathy was a frequent association (n = 37, 39.8%), and stroke was detected in a minority of cases (n = 5, 5.4%).

As previously stated, the most prevalent movement disorder was myoclonus [4,11,13,14,15,16,17,18,27,28,30,31,32,33,34,39,41,43,47,48,50,51,52], which often occurred in conjunction with encephalopathy/delirium. More than a third of the sample had ataxia [16,17,18,19,20,21,22,23,27,29,32,33,34,37,38,39,40,43,46,50,51], which often co-occurred with myoclonus. Oculomotor symptoms, such as saccadic oscillations (opsoclonus, ocular flutter), have also been documented and were often associated with ataxia [13,14,16,18,19,27,29,41,43,47,50,51]. Among the cases presenting with ataxia, a few were associated with acute polyradiculopathy/neuropathy (sensory ataxia, in the context of Miller-Fisher syndrome) [19,20,37]. In contrast, others presented cerebellar ataxia in a cerebellitis context [23] or unremarkable MRI and CSF examinations [16,17,18,21,25,27].

Not surprisingly, confusional or encephalopathic states were highly prevalent in patients with action and stimulus-sensitive myoclonus. Myoclonus is a frequent involuntary movement in critically ill patients triggered or aggravated by several different drugs or by metabolic or hypoxia-related brain dysfunction [53]. Encephalopathy and delirium are very prevalent in critical care [54], and the reviewed sample has a significant proportion of hospitalized and critical patients. Three cases of new-onset rigid-akinetic parkinsonism were described in detail [10,11,26], one of which presented with encephalopathy and generalized myoclonus [11], two of which were preceded only by a mild respiratory syndrome [10,26].

Some *de novo* movement disorders were presumably drug-induced: two serotonin syndrome cases possibly related to lopinavir/ritonavir combined with drugs with serotonergic properties, and three tardive syndrome cases (described as rigid-akinetic syndromes combined to orobuccal stereotypies) [13]. Due to incomplete information, it was not feasible to establish a causal link between myoclonus and drug administration in critically ill mechanically ventilated patients, in whom sedative agents, especially opioids, or antipsychotic medications, may have contributed to myoclonus.

### 3.3 Neuroimaging (MRI, CT, FDG-PET, and functional dopaminergic imaging)

Neuroimaging (Brain MRI or head CT) findings were reported in 82.8% (n = 77) of the 93 cases. About two-thirds (n = 56, 72.7%) of these cases demonstrated normal or non-related findings (chronic findings, such as white matter small vessel disorder). Five patients had cerebral microbleeds (6.5%). Stroke was disclosed in 4 cases (5.2%). Post-contrast focal enhancement or edema was seen in 10 cases (12.9%). Some of these observations are described in greater detail below: Gadolinium (Gd) enhancement of the oculomotor nerve (in a Miller-Fisher syndrome case, 1/77, 1.3%), cerebellar edema with Gd enhancement (1/77, 1.3%), cortical or brainstem hypoxic-ischemic lesions (2/77, 2.6%), pachymeningeal enhancement (1/77, 1.3%), acute leukoencephalitis (3/77, 3.9%), splenium DWI hyperintensity (1/77, 1.3%), splenium microbleeds (1/77, 1.3%), fornix edema (1/77, 1.3%), orbitofrontal hyperintensity (1/77, 1.3%), middle cerebral artery stroke (2, 2.6%), and multiple focal enhancing lesions in putamen and cerebellum (1, 1.3%).

FDG-PET scan was performed in five patients, as described below. It showed normal glucose metabolism in a case with parkinsonism [10]. Two of these five scanned patients had myoclonus and ataxia [34,43], one had ataxia and other cerebellar signs [43], and one had orofacial myoclonus [47]. FDG-PET revealed hypermetabolic cerebellum in all four cases: two also had hypermetabolism in the basal ganglia, while the other three had cortical hypometabolism.

The three parkinsonism cases had confirmed nigrostriatal denervation by functional dopaminergic imaging, using 18F-DOPA PET, DAT-Scan, or TRODAT-1 SPECT.

### 3.4 Cerebrospinal fluid analysis

For 48.4% (n = 45) of the examined cases, cerebrospinal fluid (CSF) analysis was available. About 73.3 % (n = 33) of the patients had a normal cell count, while 26.7% (n = 12) had mild-to-moderate pleocytosis. We observed that 51% (n = 23) of patients had normal CSF protein levels, while 49.9% (n = 22) had slightly elevated protein levels.

Antineuronal paraneoplastic antibodies (Abs) were not detected in any of the cases when a standardized Abs panel was tested (n = 9). Antibodies against Purkinje cells, striatal, and hippocampal neurons were observed in one patient with subacute cerebellar syndrome and myoclonus utilizing tissue immunostaining [34]. Franke et al. used indirect immunofluorescence to screen antibodies against novel CNS epitopes in unfixed mouse brain sections. They found staining associated with vessel endothelium, astrocytic proteins, and neuropil of basal ganglia, hippocampus, or olfactory bulb [47].

SARS-CoV-2 RT-qPCR positivity in CSF samples appears to be uncommon: it was detected in only one case of cerebellitis [23].

### 3.5 Neurophysiological techniques (EEG and EMG)

EEG findings were available in 29% (n = 27) of the cases. Of these, 59.3% (n = 16) had background slowing, while 33.3% (n = 9) had normal results. In one case, lateralized periodic discharges were observed in association with diaphragmatic myoclonus [31], while in another, frontal spikes were observed [21]. Two studies (three cases) used neurophysiological measures to characterize the possible CNS topographic origin of myoclonus [15,31], using a combination of EEG and EMG or jerk-locked registers. Two of the three cases had cortical/subcortical origin, and one, a subcortical source.

### 3.6 Treatment attempts

In 39.8% of the cases (n = 37), the information about treatment attempts was incomplete or missing. There was immunotherapy with oral/intravenous steroids (n = 21, 37.5%). In 11 cases, the corticosteroid therapy targeted the movement disorder; in 5 cases, it targeted COVID-19-related inflammatory reaction and, in 4 cases, the target was not clear. Immunoglobulin (IVIg) was used in 32.14% (n = 18) of the reported cases with available treatment information (n = 56). In all of these cases, IVIg therapy was targeting the movement disorder. Plasma exchange was tried in one case. Immunotherapy was used primarily in inpatient settings, but cases ranged from mild to critically ill in mechanical ventilation.

Antiepileptic drugs and benzodiazepines such as clonazepam (n = 17), levetiracetam (n = 16), valproic acid (n = 10), and lorazepam (n = 5) seemed to be favored for symptomatic care, mostly for the myoclonus presentation. Additionally, dexmedetomidine (n = 5), ketamine (n = 1), primidone (n = 1), and midazolam (n = 1) were used in treatment attempts.

### 3.7 Bayesian regression using zero-inflated Poisson distribution

In this analysis, we generated a regression using Bayesian zero-inflated Poisson (ZIP), a model for counts-based datasets with an excess of zero-valued data in the dependent variable (assuming a constant zero-inflation probability across observations). A value of “zero” indicates that nothing occurred, which may be because the events’ rate is low or because the process that causes events failed to start.

**Figure 4:**
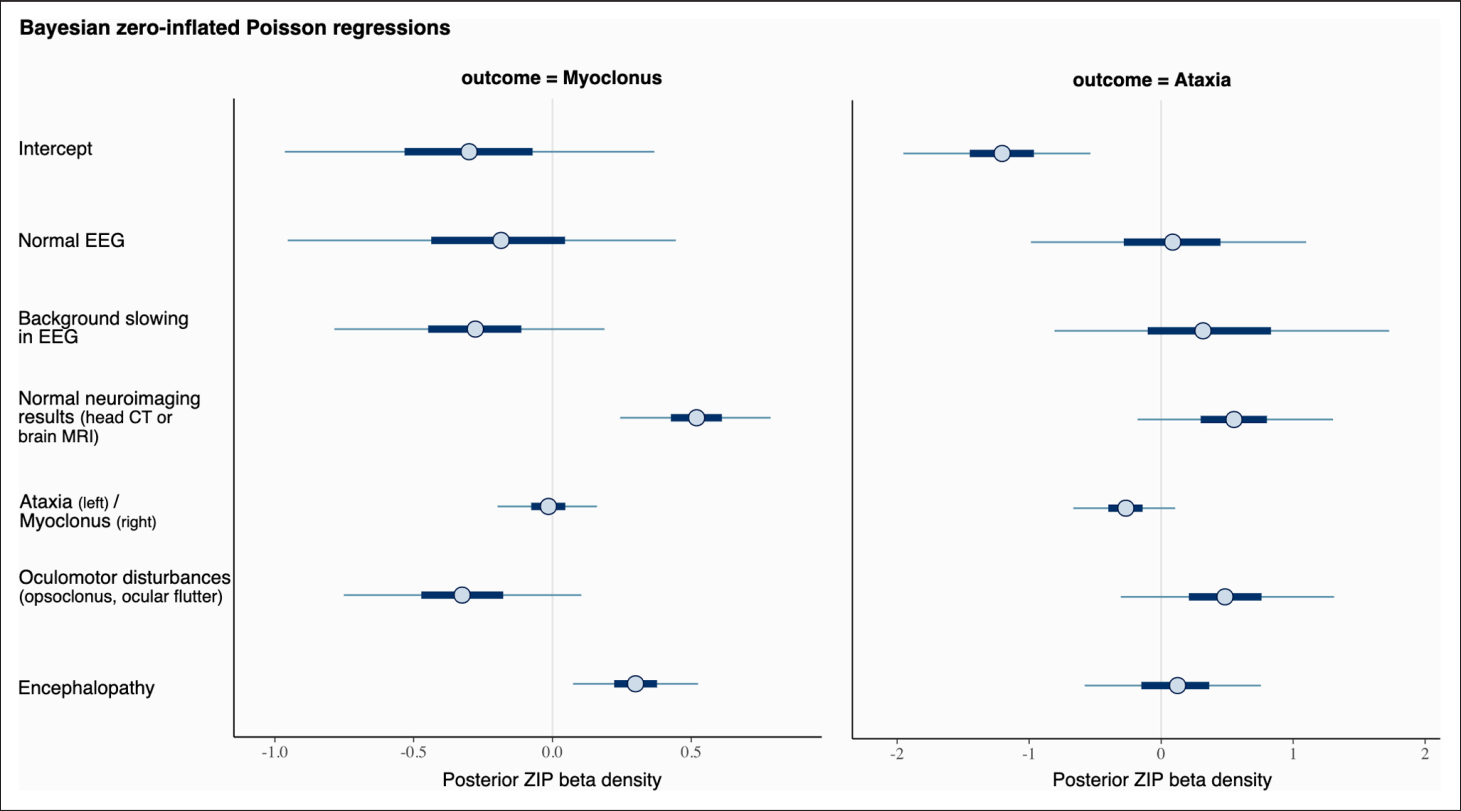
Posterior beta densities from the Bayesian zero-inflated Poisson (ZIP) regressions. The thick segments indicate 50% intervals, while the thinner outer lines indicate 90% intervals. The points in the above plot indicate the posterior medians. Data source: extracted from individual cases, original articles.

Within the data extracted from the sample of COVID-19 associated with new-onset movement disorders, myoclonus was associated with normal neuroimaging results (head CT or brain MRI) and encephalopathy (left side of ***Figure 4***), where the posterior densities of the beta distributions (obtained from the Bayesian zero-inflated Poisson regression) do not exceed zero (central line). When ataxia was used as the outcome variable (right side of ***Figure 4***), no relation was observed (all posterior beta densities crossed zero) with the dependent variables.

### 3.8 Distinct syndromes associated with myoclonus

Cluster analysis was utilized to infer movement disorders syndromic grouping from the reviewed articles. According to a seven indices ensembling method (NbClust), we found two as the optimal number of clusters. The PAM method then evaluated dissimilarity among objects. By assembling two unique groups, the random forest (RF) clustering approach [53] identified 36 articles with a higher proportion of myoclonus reports associated with encephalopathy and eight articles with a higher proportion of myoclonus connected with ataxia or oculomotor disturbances.

We interpreted these findings as suggestive of at least two myoclonus-associated syndromes that might occur in COVID-19: (1) a syndrome that associates myoclonus and encephalopathic state (which, we speculate, may relate to with broader brain dysfunction and neuronal hyperexcitability due to clinical complications in critical care or pharmacological interactions); and (2) a post-viral syndrome with an autoimmune origin that links oculomotor disturbances, ataxia, and myoclonus, resembling an opsoclonus-myoclonus-ataxia syndrome.

## 4. Discussion

Notably, myoclonus is the most frequently identified movement disorder associated with COVID-19. Myoclonus is defined as a sudden, brief involuntary muscle contraction (positive myoclonus) or abrupt cessation of muscle contraction (negative myoclonus) [55]. It occurs in a wide variety of etiologies, including post anoxic (Lance-Adams syndrome), metabolic (liver and renal failure, electrolyte imbalances), toxic, drug-induced (opioids, levodopa, antidepressants, quinolones, antiepileptic drugs), paraneoplastic, autoimmune (as in opsoclonus-myoclonus-ataxia syndrome), infectious (or postinfectious), genetic and neurodegenerative disorders. Additionally, it can be categorized into cortical, subcortical, spinal, or peripheral myoclonus based on its underlying neurophysiology (CNS source) [55].

It is critical to emphasize that in cases stated to be associated with COVID-19, several factors can contribute to the incidence of myoclonus: kidney failure, usage of provoking drugs in the context of intensive care, such as antibiotics, fentanyl, propofol, or phenytoin, as well as prolonged and sustained hypoxia [33,56].

Muccioli et al. conducted a comprehensive clinical and paraclinical assessment, suggesting that myoclonus could have a subcortical origin [30]. Interestingly, a proportion of myoclonus cases seemed to present in a delayed-onset compared to the infectious syndrome [28,29,30]; concurrent ataxic manifestations were not unusual. Recent publications have emphasized the description of opsoclonus-myoclonus-ataxia (in its complete or incomplete clinical syndrome) and included clinical improvement after IVIg therapy [27,50,51]. Myoclonic jerks were usually symptomatically treated with antiepileptic drugs (levetiracetam and valproate) and benzodiazepines (clonazepam, lorazepam).

According to a published comment on this topic, myoclonus’s underlying pathophysiology in COVID-19 may have a post-hypoxic or a postinfectious nature [56]. Although a putative physiopathology involving antigenic cross-reactivity with neuronal proteins from the brainstem or cerebellum remains hypothetical, an interesting case series from Germany provides initial support for this thesis [47]. The CNS’s postulated role in the production of myoclonus could be attributed to brainstem hyperexcitability or even lack of cerebellar inhibitory output [56]. Normal structural neuroimaging findings are consistent with the hypothesis that some of these movement disorders may be mediated by post-viral immune processes rather than structural lesions.

In a small number of cases, the CSF profile was examined. A few patients presented with mild mononuclear pleocytosis. The most frequently observed phenotype was normal cell count, mild hyperproteinemia, and normal glucose levels. The rise in CSF protein levels may be caused by blood-CSF barrier disruption, intrathecal immunoglobulin synthesis, or even be secondary to pre-existing medical comorbidities. However, further research is needed to ascertain an intrathecal immunological or parainfectious reaction, using techniques such as Reiber diagram and comprehensive autoantibodies analysis [57].

FDG-PET results provide some insights: they indicate that in some cases, presenting as movement disorders, an active ongoing metabolic process involving primarily the cerebellum and basal ganglia is implicated [34]. Additionally, they support a subcortical origin for myoclonus in COVID-19. Further research is required to validate this idea and better understand these metabolic PET patterns [32].

Parkinsonism related to SARS-CoV2 seems to be a very unusual manifestation. Brundin et al. hypothesized that parkinsonism links with COVID-19 through the following mechanisms: (a) vascular insults to the nigrostriatal system (if with imaging compatible with stroke); (b) neuroinflammation triggered by systemic inflammation; (c) neuroinvasion and direct neuronal damage by the virus, that could gain access to the brain via olfactory nerve or gastrointestinal/respiratory tract via the vagus nerve [58].

The three initial cases linking COVID-19 and parkinsonism had acutely developed severe symptoms, which could suggest a direct link between nigrostriatal damage and SARS-CoV-2 but are not indeed able to prove a causal relationship between COVID-19 and Parkinson’s disease [10,11,26]. Prodromal or presymptomatic PD could be unmasked when individuals are acutely ill. We could not reject that this might be the case in the reported patients with “acute onset” parkinsonism. Cohort studies of COVID-19 persistent anosmic cases, followed up for years, will help to disentangle such a link.

Serotonin syndrome was documented in two patients due to combined drugs for COVID-19 and other comorbidities [11]. Due to the high prevalence of psychiatric disorders, it is critical to consider this a possible differential diagnosis when myoclonus is between the clinical signs, as it is a treatable condition.

Neuropathological assessment of COVID-19 came initially from small case series that described hypoxic injury, blood-brain barrier breakdown, microscopic infarcts, hemorrhagic and demyelinating white matter lesions, and a scarcity of RT-qPCR positivity in brain samples [59,60,61]. Microglial activation, microglial nodules, and neuronophagia have also been described [62]. To our knowledge, no neuropathological study has been conducted to precisely examine the relationship between SARS-CoV-2 and movement disorders during acute or post-acute infection.

Our study has several limitations: heterogeneity of the sample (with a predominance of case reports and case series), a bias towards hospitalized patients with severe COVID-19 (requiring consultation with a neurology specialist), heterogeneity of available information, lack of a control group (composed of patients with similar infectious conditions, for example) for prevalence rates comparison. Even with a standardized data collection protocol, we could not address the issue of missing information. Thus, these findings produce hypotheses for future research rather than lead to causal inferences or overinterpretation.

Movement disorders were early (in the pandemic’s course) predicted to manifest in COVID-19 patients. However, they represent uncommon or underreported phenomena concerning the infected population as a whole [63]. This brief study contributes to the clinical scenario of SARS-CoV-2. It guides general practicing neurologists to the potential identification of these uncommon movement disorders syndromes as part of COVID-19 symptomatology or a consequence of its pharmacological management or clinical complications. Myoclonus and ataxia deserve special attention in upcoming studies: these two manifestations were the most prevalent in the reviewed data, could appear in combination, and may have an immune origin. The direct link between movement disorders and SARS-CoV-2 (and its underlying mechanisms) will be established or disproved in still lacking comprehensive neurophysiological, molecular, pathological, and neuroimaging studies with large samples.

## Additional File

The additional file for this article can be found as follows:

10.5334/tohm.595.s1 Supplementary Figure 1. Optimal number of clusters obtained through seven distinct clustering indexes, using NbClust ensemble method.
