# Supplementary material for: New-Onset Movement Disorders Associated with COVID-19
Source: Tremor Other Hyperkinet Mov (N Y). 2021 Jul 8;11:26. doi: 10.5334/tohm.595 (PMC8269765; doi:10.5334/tohm.595)
Supplement: Supplementary Figure 1. — Optimal number of clusters obtained through seven distinct clustering indexes, using NbClust ensemble method. [file tohm-11-1-595-s1.pdf]

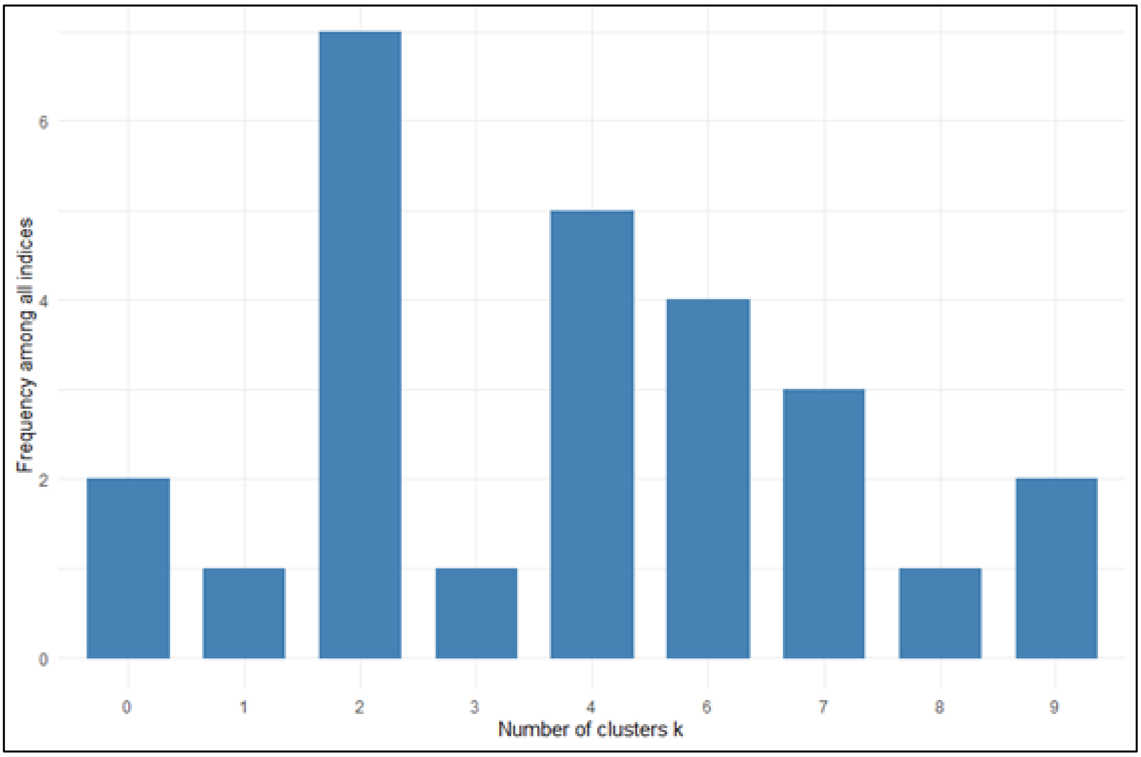

Supplementary Figure 1. Optimal number of clusters obtained through seven distinct clustering indexes, using NbClust ensemble method
